# Editorial Note: Sanitation facilities, hygienic conditions, and prevalence of acute diarrhea among under-five children in slums of Addis Ababa, Ethiopia: Baseline survey of a longitudinal study

**DOI:** 10.1371/journal.pone.0329217

**Published:** 2025-07-29

**Authors:** The PLOS One Editors

This article [1] was identified as one of a series of articles for which the *PLOS One* Editors have concerns about authorship and adherence to the journal’s first and second publication criteria. We regret that the issues were not addressed prior to the article’s publication.
